# Toxic effects of titanium dioxide nanoparticles on reproduction in mammals

**DOI:** 10.3389/fbioe.2023.1183592

**Published:** 2023-05-12

**Authors:** Fan Minghui, Sun Ran, Jiang Yuxue, Sheng Minjia

**Affiliations:** The Reproductive Medical Center, China-Japan Union Hospital of Jilin University, Changchun, China

**Keywords:** titanium dioxide nanoparticles, nano-TiO2, reproductive, toxicty, mammal

## Abstract

Titanium dioxide nanoparticles (nano-TiO_2_) are widely used in food, textiles, coatings and personal care products; however, they cause environmental and health concerns. Nano-TiO_2_ can accumulate in the reproductive organs of mammals in different ways, affect the development of the ovum and sperm, damage reproductive organs and harm the growth and development of offspring. The oxidative stress response in germ cells, irregular cell apoptosis, inflammation, genotoxicity and hormone synthesis disorder are the main mechanisms of nano-TiO_2_ toxicity. Possible measures to reduce the harmful effects of nano-TiO_2_ on humans and nontarget organisms have emerged as an underexplored topic requiring further investigation.

## 1 Introduction

Nanomaterials, as a kind of ultrafine particle material, have the characteristics of the nanosize effect, the surface effect and the macroscopic quantum tunnelling effect. Nanomaterials are widely used in biological engineering, the medical field, ceramics, cosmetics, electronic sensors and other fields; nanomaterials include nanoparticle materials, nanomagnetic liquid materials, nanosolid materials, and nanofilm materials (Chen et al., 2020). Although nanomaterials are widely used, the toxicity studies of nanomaterials are not comprehensive and in depth. Titanium dioxide nanoparticles (nano-TiO_2_, <100 nm) are widely used in technology, industry, and consumer products (Luo et al., 2020; Cornu et al., 2022) due to their desirable physicochemical characteristics, including high reactivity, ultraviolet (UV) shielding function, large specific surface area, photocatalytic activity and unique quantum and electron-tunnelling effects (Ali et al., 2018). However, subsequent studies found that exposure to high levels of nano-TiO_2_ caused lung tumours in rats (Shi et al., 2013), and the International Agency for Research on Cancer (IARC) classified titanium dioxide as a Group 2B carcinogen (suspected carcinogen). In 2022, the European Commission (EC) clarified the definition of nanomaterials to support and align legislation across all sectors in a new recommendation. The use of TiO_2_ as a food additive was recently deemed unsafe by the European Food Safety Authority (EFSA) (Additives et al., 2021), and the EC announced the decision to ban its use. Therefore, the biological toxicity of nano-titanium dioxide has been a concern. A large number of studies have shown that after inhalation or ingestion of nano-TiO_2_, it accumulates in the lungs, digestive tract, heart, liver, kidney, spleen and reproductive organs, with different effects on the various organs (Gojznikar et al., 2022).

TiO_2_ is a natural oxide of titanium metal with a diameter of less than 100 mm. It has high thermal stability, hydrophilicity and chemical stability, as well as low toxicity and few biological effects. Brookite, anatase and rutile are the main polymorphs of nano-TiO_2_. A commonly noted property of nano-TiO_2_ is its photocatalytic ability, enabling it to stimulate the generation of free radicals, which can then react further with other compounds (Noman et al., 2019). Therefore, as a new green and efficient photocatalytic material, nano-TiO_2_ is widely used in every aspect of daily life, such as air purification, sewage treatment, and sterilization during environmental remediation. Nano-TiO_2_ is also added to cosmetics, toothpaste, ceramics and food additives. The wide application of nano-TiO_2_ increases human exposure, mainly through the respiratory tract, oesophagus and skin into the human body; furthermore, nano-TiO_2_ also accumulates in tissues and organs along with the circulatory system. Therefore, nano-TiO_2_ can affect human health through occupational exposure and the use of nano-TiO_2_-containing products directly and through environmental exposure to unintentionally released nano-TiO_2_ indirectly (Shi et al., 2013; Cornu et al., 2022). This review focuses on the effects of nano-TiO_2_ on the mammalian reproductive system.

## 2 Application of nano-TiO_2_ in medical diagnosis and treatment

Nano-TiO_2_ is used as a photosensitizer in cancer therapy and for photodynamic inactivation of antibiotic-resistant bacteria. This is possible because these nanoparticles have high biocompatibility and excellent photochemical properties. In photodynamic therapy, nano-TiO_2_ photosensitizers can be activated to produce cytotoxic ROS in response to specific wavelengths of light, thus killing tumour cells. Although UV-activated titanium dioxide nanoparticles have prospects for PDT therapy, this strategy appears to be ineffective in treating certain types of cancer and has limited clinical application. Limited UV penetration limits the technique to surface cancers such as skin cancer, nasopharyngeal cancer and oral cancer. At the same time, the duration of UV-mediated ROS production is not long enough to provide continuous and long-term anticancer effects. This limitation has led to the creation of composites containing nano-TiO_2_. The combinations of nano-TiO_2_ with carbon-based nanomaterials and inorganic dopants were studied for anticancer and antimicrobial PDT (Ni et al., 2017). Nano-TiO_2_ was applied *inter alia* in the synthesis of bioconjugates with cell-specific monoclonal antibodies to treat malignant tumours, while in the antimicrobial therapy it can be prepared of black nano-TiO_2_. Another application of nano-TiO_2_ is as a drug carrier (Liu et al., 2022). It allows drugs to reach diseased areas of the body while keeping healthy tissues unharmed (Gao et al., 2016). Antibodies or markers can be labelled on the surface of nano-TiO2 to design drug delivery to selected, diseased areas (Ghaderi et al., 2011; Jia and Jia, 2012). The proper drug delivery systems used for photosensitizers allows PDT to be performed in specific tissues. As a result, much attention should be paid to minimizing side effects and developing novel formulations that allow the direct delivery of active substances to target cells.

## 3 Effect of nano-TiO_2_ on the reproductive system

### 3.1 Accumulation of nano-TiO_2_ in genital organs

Nano-TiO_2_ can be ingested in a variety of ways and accumulates mainly in the lungs, liver, spleen, kidney, nervous system and other organs, as well as in the genital organs. In recent studies, mice were subjected to long-term exposure to nano-TiO_2_ through intragastric administration. At the end of exposure, pathological observation and Raman spectrum identification were performed, and the accumulation of Ti was detected in the ovaries of female mice (Hong et al., 2017) and the testes of male mice. This indicates that nano-TiO_2_ can penetrate the blood-testis barrier to cross into the testicular tissue and impair testicular function. Hong et al. investigated that maternal exposure to nano-TiO_2_ affect foetal development. The study shows that Ti concentrations were increased in maternal serum, placenta, and foetus in nano-TiO_2_-exposed mice. Furthermore, the number of both dead foetuses and foetuses were increased caused by Ti that were resorbed (Hong et al., 2017). Kyjovska et al. investigated the effect of maternal airway exposure to nano-TiO_2_ on the function of male reproductive system in the two following generations. Maternal exposure of nano-TiO_2_ tended to reduce sperm counts, although did not affect daily sperm production (DSP) significantly in the F1 generation. Overall, the time-to-first F2 litter increased with decreasing sperm production (Kyjovska et al., 2013). These studies demonstrated that nano-TiO_2_ can penetrate the placental barrier, as well as the blood-testis barrier. However, the transport mechanism of nano-TiO_2_ penetrating the blood-testis barrier and placental barrier remains unclear.

### 3.2 Effect of nano-TiO_2_ on the female reproductive system

Nano-TiO_2_ has been shown to accumulate in the ovaries, but it has been relatively poorly studied in female mammals. Studies have shown that the body weight, ovarian weight and ovarian index of female mice were significantly decreased after long-term exposure to low-dose nano-TiO_2_(Zhao et al., 2013; Zhou et al., 2019). Several pathological changes were observed in the nano-TiO_2_ group, including reduction in the number of ovarian follicles, ovarian cyst formation, and follicle development impairment, suggesting that ovaries were damaged by nano-TiO_2_ exposure (Karimipour et al., 2018; Zhou et al., 2019). Karimipour. et al. found that nano-TiO_2_ caused a significant reduction in oocyte number, fertilization rate, preimplantation embryo development, pregnancy rates and number of births (Karimipour et al., 2018). Recent studies have demonstrated that chronic exposure to nano-TiO_2_ resulted in a reduction in fertility and follicle development. Follicle development and fertility are associated with the levels of sex hormones. The present study demonstrated that exposure to nano-TiO_2_ significantly decreased the serum levels of progesterone (P) and testosterone (T) and increased the concentration of estradiol (E_2_) (Gao et al., 2012; Zhao et al., 2013; Tassinari et al., 2014; Karimipour et al., 2018). However, the effects of nano-TiO_2_ on the levels of follicle stimulating hormone (FSH) and luteinizing hormone (LH) are controversial. Gao et al. found that after 90 days of exposure to nano-TiO_2_ in mice, serum FSH levels increased, LH levels decreased, and prolactin (PRL) and sexual hormone binding globulin (SHBG) levels did not change significantly (Gao et al., 2012). Zhao et al. found that under the same exposure, both serum FSH and LH levels in mice decreased significantly (Zhao et al., 2013). Interestingly, in the latest study, Ji et al. found that the serum levels of FSH and LH were significantly increased in mice exposed to the same dose of nano-TiO_2_ for 60 days (Ji et al., 2023). The different results may be related to the species line of experimental animals, the dose of nano-TiO_2_, exposure time, detection method, and the mall sample size, which need to be verified by further studies. Progesterone release was diminished after the addition of nano-TiO_2_ during porcine granulosa cell incubation (Sirotkin et al., 2021a; Sirotkin et al., 2021b). Nano-TiO_2_ may cause the disturbance of steroidogenesis, which results in a reduction in fertility and follicle development. The basic details of the involved studies are shown in Table 1.

**TABLE 1:** Studies of nano-TiO2 on the female reproductive system.

Authors	Year	Organ	Characteristic of nano-TiO_2_	Materials	Method	Results				References
						Weight	Histopathologic change and other results	Hormone	Mechanism	
Gao et al.	2012	ovary	Anatase;	150 ICR mice (23 ± 2 g)	Intragastric, 2.5, 5, and 10 mg/kg BW for 90 days	-	Mitochondrial swelling and cristae breakage, nucleus chromatin condensation and margination, and irregularity of the nuclear membrane in ovarian cells	E_2_, FSH↑ P, LH, T↓	Ovarian apoptosis Increased expression of Cyp17a1, Akr1c18	Gao et al. (2012)
Zhao et al.	2013	ovary	Anatase	400 ICR mice (18 ± 2 g)	Intragastric, 2.5, 5, and 10 mg/kg BW for 90 days	BW↓	A large of atretic follicles, severe inflammatory cell infiltration, and necrosis, black agglomerates in the ovary	E_2_↑,	IGFBP-2, EGF, TNF-a, tPA, IL-1b, IL-6, Fas, and FasL expression, while decreased IGF-1, LHR, INH-a, and GDF-9 expression	Zhao et al. (2013)
								FSH, P, LH, T↓		
								PRL, SHBG -		
Tassinari et al.	2014	ovary	Anatase;	42 SD rat aged 60 days	Intragastric, 1, 2 mg/kg BW for 5 days	-	Apoptosis in granulosa cells	T↓	Apoptosis in granulosa cells	Tassinari et al. (2014)
			<25 nm							
			20–60 nm							
Karimipour et al.	2018	ovary	10–25 nm	54 NMRI mice aged 10 weeks	Intragastric, 100 mg/kg/d BW for 5 days	-	Ovarian follicles↓	E_2_↑	Oxidative stress: MDA↑	Karimipour et al. (2018)
							Ovarian cyst formation			
							Oocyte number, fertilization rate, and preimplantation embryo development↓			
Hong. et al.	2018	ovary	Anatase	200 SPF female mice aged 4 weeks	Intragastric, 2.5,5 and 10 mg/kg/d BW for 30 days	BW, Ovary weight↓	Inflammatory cell infiltration, increased primary atretic follicle and	E2,P,AMH, Inhibin B↓ FSH,LH,TSHFSH/LH↑		Hong and Wang (2018)
							apoptosis of granule cells			
							Mating and pregnancy rates ↓			
Zhou. et al.	2019	ovary	6–7 mm	80 ICR mice (20 ± 2 g)	Intragastric, 1.25, 2.5, and 5 mg/kg BW for 60 days	Ovary weight↓	The number of primordial, secondary, and antral follicles and corpus luteum↓ The number of atretic follicles↑	-	Dysfunction of the TGF-β, PI3K/AKT/mTOR, and AKT/p70S6K-rpS6/TSC/mTOR pathways	Zhou et al. (2019)
Sirotkin et al.	2021	GCs	mixture of rutile and anatase, <100 nm	Porcine granulosa cells	0, 0.01, 0.1, 1 or 10 μg/mL	-	-	P↓	Expression of mRNAs for proliferating cell nuclear antigen (PCNA), cyclin B1, bax and caspase 3↓	Sirotkin et al. (2021a)
Sirotkin et al.	2021	GCs	mixture of rutile and anatase, <100 nm	Porcine granulosa cells	0.1, 1, 10 or 100 μg/mL for 2 days	-	-	P↓	-	Sirotkin et al. (2021b)
Ji et al.	2023	ovary	-	Female mice	2.5, 5, or 10 mg/kg for 60 days	-	-	FSH, LH↑	Expression of activin, follistatin, BMP2, BMP4, TGF-β1, Smad2, Smad3, and Smad4↑	Ji et al. (2023)
									Inhibin-α expression↓	

### 3.3 Effect of nano-TiO_2_ on the male reproductive system

Nano-TiO_2_ can accumulate in the testis through the blood-testis barrier and have side effects on the male reproductive system. However, the specific mechanism remains unknown. Santonastaso et al. provided data on the evaluation of the potential genotoxicity of nano-TiO_2_ *in vitro* on human sperm cells. The results showed that nano-TiO_2_ can reduce sperm motility, induce the loss of sperm DNA integrity, cause sperm DNA fragmentation, decrease sperm genomic stability and increase intracellular reactive oxygen species (ROS) in sperm cells (Santonastaso et al., 2019). In animal experiments, the body weight, testicular weight and relative testicular weight decreased significantly after intragastric exposure to nano-TiO_2_, and the reduction was dose-dependent (Gao et al., 2013; Jia et al., 2014; Orazizadeh et al., 2014; Hong et al., 2015a; Hong et al., 2016a; Shahin and Mohamed, 2017; Lauvas et al., 2019; Meng et al., 2022; Li et al., 2023). Shahin et al. found that the weight of the rats prostates decreased significantly after exposure to nano-TiO_2_(Shahin and Mohamed, 2017). Several studies have demonstrated that nano-TiO_2_-treated rats clearly exhibited loss of normal architecture, degeneration of the seminiferous tubules, reduction in the number of spermatogenic cells, and infiltration of inflammatory cells in the testis (Meena et al., 2015; Hong et al., 2016a). Nano-TiO_2_ could migrate to Sertoli cells (SCs) and Leydig cells (LCs), which induced intracellular vacuoles, endoplasmic reticulum dilation, mitochondrial oedema, and chromatin distribution abnormalities in spermatogenic cells (Jia et al., 2014; Orazizadeh et al., 2014; Hong et al., 2015b; Hong et al., 2016a; Hong et al., 2016b; Shahin and Mohamed, 2017; Hussein et al., 2019; Lauvas et al., 2019; Ogunsuyi et al., 2020).

Nano-TiO_2_ affects the parameters of sperm in males. A number of *in vivo* studies in mice or rats demonstrated that nano-TiO_2_ is able to cross the blood-testis barrier and accumulate in the testis, resulting in a reduction in sperm numbers and motility and an increase in sperm morphological abnormalities, resulting in a reduction in the mating rate, fertility and number of offspring (Komatsu et al., 2008; Guo et al., 2009; Gao et al., 2013; Orazizadeh et al., 2014; Hong et al., 2015a; Hong et al., 2015b; Hong et al., 2016a; Khorsandi et al., 2017; Miura et al., 2017; Morgan et al., 2017; Shahin and Mohamed, 2017; Hussein et al., 2019; Lauvas et al., 2019; Miura et al., 2019; Santonastaso et al., 2019; Ogunsuyi et al., 2020; Additives et al., 2021; Danafar et al., 2021; Meng et al., 2022; Li et al., 2023). This may be caused by dysfunction of steroidogenesis and spermatogenesis after nano-TiO_2_ exposure. Several studies have demonstrated a marked decrease in serum T after chronic exposure to nano-TiO_2_(Gao et al., 2013; Jia et al., 2014; Orazizadeh et al., 2014; Hong et al., 2015b; Khorsandi et al., 2017; Morgan et al., 2017; Shahin and Mohamed, 2017; Li et al., 2018; Hussein et al., 2019; Lauvas et al., 2019; Ogunsuyi et al., 2020; Danafar et al., 2021; Liu et al., 2021; Halawa et al., 2022). Li. et al. cultured primary SD rat LCs *in vitro* and exposed them to different concentrations of nano-TiO_2_ for 24 h; the result clearly showed that T production in LCs was significantly lowered following simultaneous nano-TiO_2_ treatment (Li et al., 2018). However, individual studies have not found significant effects of nano-TiO_2_ on serum T levels (Miura et al., 2017; Lauvas et al., 2019). Regarding the effects of nano-TiO_2_ on serum FSH and LH levels, the results are not consistent. Gao et al. observed that intragastric injection with 2.5, 5, and 10 mg/kg BW nano-TiO_2_ for 90 days could reduce the serum FSH and LH levels (Gao et al., 2013). Hussein et al. obtained similar results in male SD rats after 30 days of intragastric injection with 300 mg/kg BW nano-TiO_2_(Hussein et al., 2019). However, Shahin et al. found that intragastric injection with 50 mg/kg BW nano-TiO_2_ for 3 weeks could significantly increase the serum FSH and LH levels (Shahin and Mohamed, 2017). Ogunsuyi et al. treated male Swiss rats with 75 mg/kg BW nano-TiO_2_ by gavage for 35 days, and the serum FSH level increased and the LH level decreased (Orazizadeh et al., 2014). In conclusion, exposure to nano-TiO_2_ can lead to disruption of steroidogenesis and decrease serum T levels, but there is no clear result on the effects on serum FSH and LH level, which may be related to the species line of experimental animals, the dose of nano-TiO_2_, or the exposure time, which need to be verified by further studies. The basic details of the involved studies are shown in Table 2.

**TABLE 2:** Studies of nano-TiO2 on the male reproductive system.

Authors	Year	Organ	Characteristic of nano-TiO_2_	Materials	Method	Results				References
						Weight	Histopathologic change and other results	Hormone	Mechanism	
Guo et al.	2009	Testis	Anatase	45 male ICR mice aged 6 weeks	Intraperitoneally injected, 200 and 500 mg/kg	-	Sperm density and motility↓, Sperm malformation↑	-	Germ cell apoptosis	Guo et al. (2009)
Gao et al.	2013	Testis	-	115 CD-1 male mice aged 5 weeks	Intragastric, 2.5, 5, and 10 mg/kg BW for 90 days	Testicular weight↓, relative testicular weight↓, BW↓	Sperm concentration↓ Motility rate↓	FSH, LH, T↓	Spermatogenesis: upregulated: Ly6e, downregulated: Adam3, Tdrd6, Spata19, Tnp2, Prm1	Gao et al. (2013)
									Apoptosis and oxidative stress: upregulated: Axud1, Cyp1b1, Cyp2e1, Gpx5, Th	
Jia et al.	2014	Testis	Anatase; 25 nm	60 Kunming male mice aged 3 weeks	Intragastric, 10, 50 or 250 mg/kg BW for 42 days	BW↓	Vacuoles in seminiferous tubules (50, 250 mg/kg); Decreased layers of spermatogenic cells (250 mg/kg)	T↓(250 mg/kg)	Downregulated: P450-17α, 17β-HSD; Upregulated: Cyp19; No effect: P450scc, 3β-HSD, AR	Jia et al. (2014)
Orazizadeh et al.	2014	Testis	-	32 NMRI mice aged 6–8 weeks	Intragastric, 0.2, 10, 300 mg/kg BW for 35 days	Testicular weight↓, relative testicular weight↓	Sperm count, motility↓Vacuolization of the seminiferous tubules, decreased layers of spermatogenic cells	T↓	-	Orazizadeh et al. (2014)
							Johnsen’s score↓			
Meena et al.	2015	Testis	-	24 Wistar rats aged 8 weeks	Intravenous injected (through caudal vein), 5, 25, and 50 mg/kg BW for 30 days	Average coefficient of testis↓	Apoptotic cell population increased, Inflammation in testicular cells	T↓ (25 mg/kg)	Oxidative stress: SOD, GPx↓, CAT, lipid peroxidase ↑	Meena et al. (2015)
Hong et al.	2015	Testis	Anatase; 5.5 nm	160 ICR mice aged 4 weeks	Intragastric, 1.25, 2.5, or 5 mg/kg BW for 6 months	BW, testis and epididymis indices↓	Sperm count and motility↓	T↓	Expression of Cdc2, Cyclin B1, Dmcl, TERT, Tesmin, TESP-1, XPD, and XRCCI mRNA↓Gsk3-β mRNA↑ in the testicular tissues.	Hong et al. (2015b)
							Spano-sperm, sperm breakages, rarefaction of Sertoli cells and Leydig cells		Cdc2, DMC1, TERT, Tesmin, XRCC1 and XPD expressions↓, Gsk3-β and PGAM4↑	
Hong et al.	2015	Testis	Anatase; 5–6 nm	160 ICR mice aged 4 weeks	Intragastric, 2.5, 5 or 10 mg/kg BW for 60 days	BW, testicular weight, relative testicular weight↓	Sperm count and motility↓	-	LDH, SODH, SDH, G6PD, Na^+^/K^+^ -ATPase, Ca^2+^-ATPase, and Ca^2+^/Mg^2+^-ATPase ↓	Hong et al. (2015a)
							Sperm malformation↑		ACP, AKP, and NOS↑	
							Spano-sperm, sperm breakages, rarefaction of Sertoli cells and Leydig cells		Oxidative stress: ROS↑	
							Decreased layers of spermatogenic cells			
Asare et al.	2016	Testis	21 nm	6 *Ogg1* ^*−/−*^ KO mice aged 8–12 weeks, 6 Isogenic *Ogg1* ^*+/+*^ mice (WT)	Intravenous injected (tail vein),	-	-	-	DNA damage	Asare et al. (2016)
					5 mg/kg BW for 1 or 7 days				Oxidative stress in testis, Atr and Rad51, Ddb2, Sod1 and Fos expression↑	
Hong et al.	2016	Testis	Anatase; 5–6 nm	160 ICR mice aged 5 weeks	Intragastric, 1.25, 2.5, or 5 mg/kg BW for 9 months	-	Mating rate, pregnancy rate and number giving birth/foetus↓	-	The expression of Tyro3, Axl, Mer, IκB, SOCS1, and SOCS3 genes and proteins↓	Hong et al. (2016a)
							Infiltration of inflammatory cells, Sperm breakages, rarefaction, apoptosis or necrosis of spermatogenic cells and Sertoli cells, and vacuolation of seminiferous tubule			
Hong et al.	2016	SCs	Anatase; 5–6 nm	Primary cultured Sertoli cells of ICR mice	Intragastric, 5, 15, or 30 ug/mL for 24 h	-	Cell viability↑, LDH activity↑, Apoptosis of Sertoli cells↑	-	Oxidative stress: MMP↓, ROS and lipid peroxidation level↑, SOD, CAT, and GPx↓	Hong et al. (2016b)
							Vacuolization of the seminiferous tubules, nuclear shrinkage, chromatin marginalization, endoplasmic reticulum expansion, and mitochondrial swelling		Cells apoptosis: cytochrome c, caspase-3, caspase-12, Bax, GRP78, and CHOP↓, Bcl-2↑	
Khorsandi et al.	2017	Testis	-	32 NMRI mice aged 6–8 weeks	Intragastric, 300 mg/kg BW for 42 days	-	Sperm count and motility↓	T↓	Oxidative stress: MDA↑SOD and CAT↓	Khorsandi et al. (2017)
							Sperm malformation↑			
							Apoptosis index↓			
Morgan et al.	2017	Testis	-	80 albino rats (180–200 g)	Intragastric, 100 mg/kg BW for 8 weeks	-	Sperm viability↓	T↓	Oxidative stress: MDA↑GSH↓ CAT-	Morgan et al. (2017)
							Sperm malformation↑		Apoptosis↑	
							Interstitial oedema and sloughing of its germinal epithelium		Testin gene expression↑	
Mao et al.	2017	GC-2, TM4 cells	21 nm	GC-2 cells, TM4 cells	0.1, 1, 10, 100 ug/mL for 4 h	-	Microtubule network of GC-2 Cells, microtubule dynamic of GC-2 Cells, microfilamen networks of TM4 Cells, migration ability of GC-2 Cells, phagocytic activity of TM4 Cells changed	-	-	Mao et al. (2017)
Miura et al.	2017	Testis	-	C57BL/6J mice aged 8 weeks	i.v. Injection, 0.1, 1, 2, 10 mg/kg BW for 4 weeks	BW, testicular weight↓	Sperm viability↓	T (−)	-	Miura et al. (2017)
							Sperm malformation↑			
Shahin et al.	2017	Testis	Anatase; <25 nm	48 Wistar rats aged 6–7 weeks	Intragastric, 50 mg/kg BW for 1,2,3 weeks	BW↓ prostate gland weight↓	Sperm count, motility↓	T↓, E_2_, LH, FSH↑	Apoptotic changes: anti-apoptotic factor Bcl-2↓ pro-apoptotic factor Bax/Fas/Caspase-3↑	Shahin and Mohamed (2017)
							Sperm malformation↑			
							Decreased layers of spermatogenic cells			
							Johnsen’s score↓			
Ye et al.	2017	Sertoli cells	Anatase; 5–6 nm	Primary cultured SD rat Sertoli cells (aged 18 days)	5, 15, or 30 μg/mL TiO_2_NPs for 24 h	-	Nano-TiO_2_ entered the cytoplasm and cell nuclei	-	Inflammatory cytokines TNF-α, NF-κB, I-κB and IL-1β protein expression↑	Ye et al. (2017)
									p-PKC and p-p38 MAPK proteins in SCs↑	
Li et al.	2018	Leydig cells	Anatase	Primary cultured SD rat Leydig cells	0, 10, 20,40 μg/mL for 24 h	-	Nano-TiO_2_ entered the cytoplasm and cell nuclei	T↓(20,40 ug/mL)	Mitochondrial damage: MMP↓	Li et al. (2018)
									P-ERK1/2, PKA,PKC↓3βHSD, StAR, P450scc, SR-BI, and DAX1 proteins↑	
Miura et al.	2019	Testis	-	C57BL/6J mice aged 8 weeks	IT injection, 10,50 mg/kg for 1,3,9 days	-	Sperm motility↓		[^3^H]-thymidine incorporation, and ATP level↓	Miura et al. (2019)
Santonastaso et al.	2019	Human sperm	Mixture of the rutile and anatase, 21 nm	Human sperm	1,10 μg/L for 15,30,45,90 min	-	Sperm motility, sperm genomic stability↓	-	Intracellular ROS in sperm cells↑	Santonastaso et al. (2019)
							Loss of sperm DNA integrity, sperm DNA fragmentation↑			
Hussein et al.	2019	Testis	<50 nm	70 SD rats aged 6–8 weeks	Intragastric,	-	Sperm abnormalities↑	LH, FSH,T↓	Oxidative stress: L-MDA↑SOD, CAT,GSH↓	Hussein et al. (2019)
					300 mg/kg BW for 30 days		Decreased layers of spermatogenic cells		*17β-HSD* gene expression↓*Bax* gene expression↑	
Lauvås et al.	2019	Testis	Rutile, 17 nm	47 C57BL/6J BomTac mice aged 9 weeks	Intratracheally instilled, 63 µg for 7 weeks	-	Sperm counts (−)	T (−)	-	Lauvas et al. (2019)
Ogunsuyi et al.	2020	Testis	Anatase; <25 nm	25 Swiss mice aged 11–15 weeks	Intraperitoneally injection, 9.38, 18.75, 37.5, 75 mg/kg BW for 35 days	BW, testicular weight (−)	Sperm count, motility↓	T, LH↓FSH↑	Oxidative stress: SOD↓, CAT↓, GSH↓	Ogunsuyi et al. (2020)
							Sperm malformation↑			
							Vacuolization of the seminiferous tubules, decreased layers of spermatogenic cells			
Liu et al.	2021	Testis	Anatase and rutile, 40 nm ± 5 nm	15 SD rats aged 8 weeks	Intragastric, 500 mg/kg BW for 3 and 7 days	-	Sperm counts↓	T↓	Inhibit testosterone synthesis, which is related to the reactive oxygen species (ROS)-MAPK (ERK1/2)-StAR signal pathway	Liu et al. (2021)
							Anatase is more toxic than rutile.			
Mancuso et al.	2022	Sertoli cells	-	Porcine prepubertal Sertoli cells (SCs)	5,100 μg/mL for 24 h and 1,3 weeks	-	Cell count↓	-	Apoptosis in SCs, intracellular ROS Production and DNA Damage, Stimulated proinflammatory and immunomodulatory responses, activated MAPK and NF-κB signalling pathway	Mancuso et al. (2021)
							Deeply invaginated and shrunk nuclei, disorder of chromatin components, missing endoplasmic reticulum membranes, mitochondria number↓			
							Apoptotic mitochondria, enlarged endoplasmic reticulum, increased frequency of lipid droplets			
Meng et al.	2022	Testis	Anatase; 5–10 nm	96 ICR mice aged 6–8 weeks	Intragastric, 50 mg/kg BW for 30 days	BW↓	Sperm count and motility↓	-	Oxidative stress: SOD↓, MDA↑	Meng et al. (2022)
							Sperm malformation↑		Germ cell apoptosis by inhibiting mitochondrial apoptotic pathway, expression of Bcl-2↑, Bax, Cleaved Caspase 3, and Cleaved Caspase 9↓	
							Vacuolization of the seminiferous tubules, decreased layers of spermatogenic cells			
Danafar et al.	2022	Testis	-	32 NMRI mice aged 8–12 weeks	Intragastric, 2.5,5,10 mg/kg BW TiO_2_NPs for 40 days	-	Sperm malformation↑, spermatogenesis index and lumen parameters↓	T↓,	Malondyaldehyde in the seminal fluid↑ MDA↑	Danafar et al. (2021)
							Leydig cell count↓	FSH, LH, E2 (−)		
							decreased layers of spermatogenic cells			
Li et al.	2023	Testis	-	32SD rats aged 8 weeks	Intragastric, 50 mg/kg BW for 90 days		Sperm count, mobility↓		Oxidative stress: GSH-Px, CAT↓, MDA, LDH↑	Li et al. (2023)
							Sperm malformation↑		Testicular cell apoptosis, DNA damage in sperm	
Halawa et al.	2022	Testis	50–55 nm	20 Albino rats aged 3–4 months	150 mg/kg BW for 14 days	Testicular weight (−)	Decreased layers of spermatogenic cells, vacuolization of the seminiferous tubules, infiltration of inflammatory cells	T↓	Oxidative stress: GSH,GST,SOD,CAT,GPx, GSH-Px↓MDA↑	Halawa et al. (2022)

## 4 Mechanism of reproductive toxicity of nano-TiO_2_

### 4.1 Oxidative stress

**FIGURE 1:**
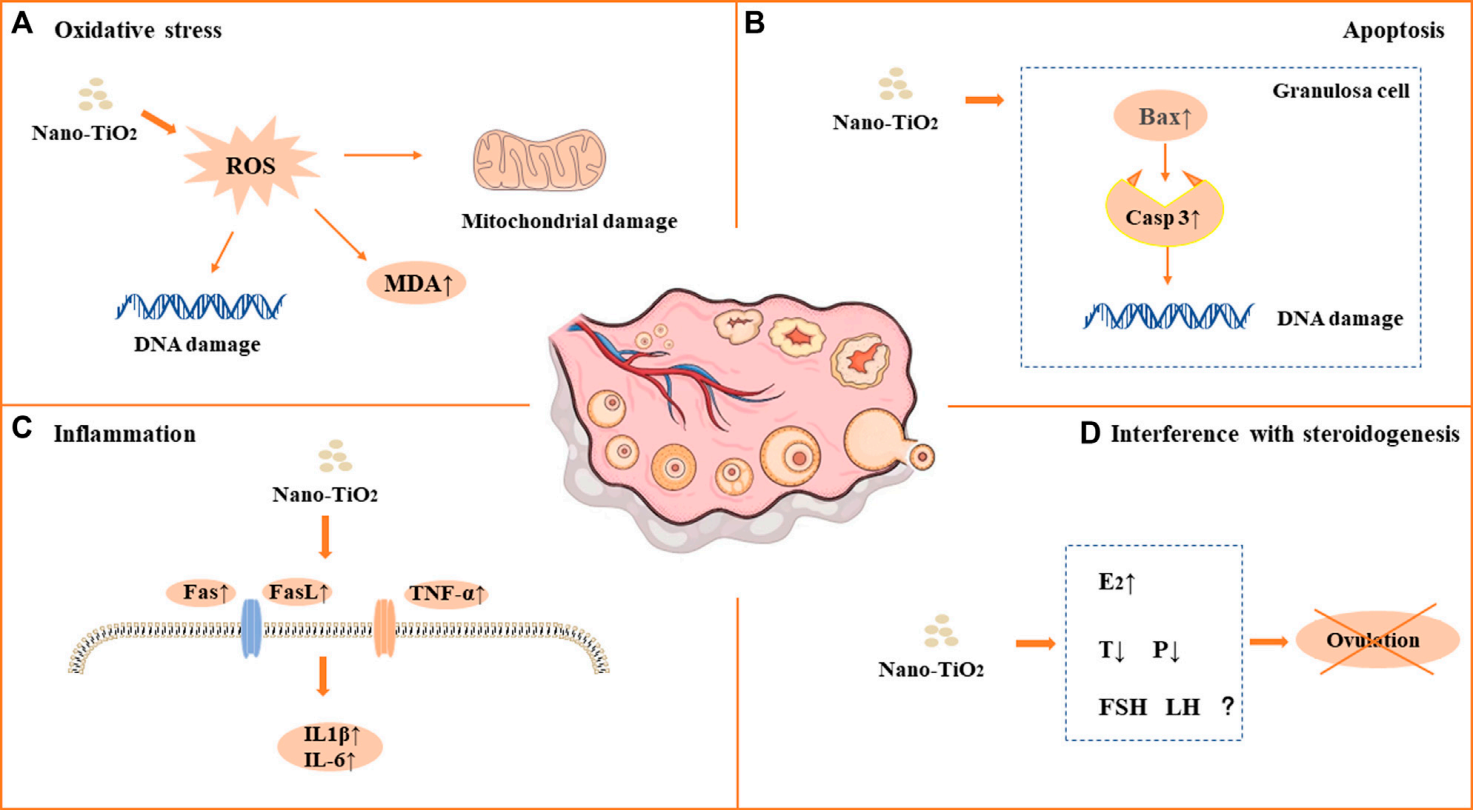
Mechanism of toxicity of nano-TiO_2_ on reproduction in female mammals. **(A)** Oxidative stress. **(B)** Apoptosis. **(C)** Inflammation. **(D)** Interference with steroidogenesis.

**FIGURE 2:**
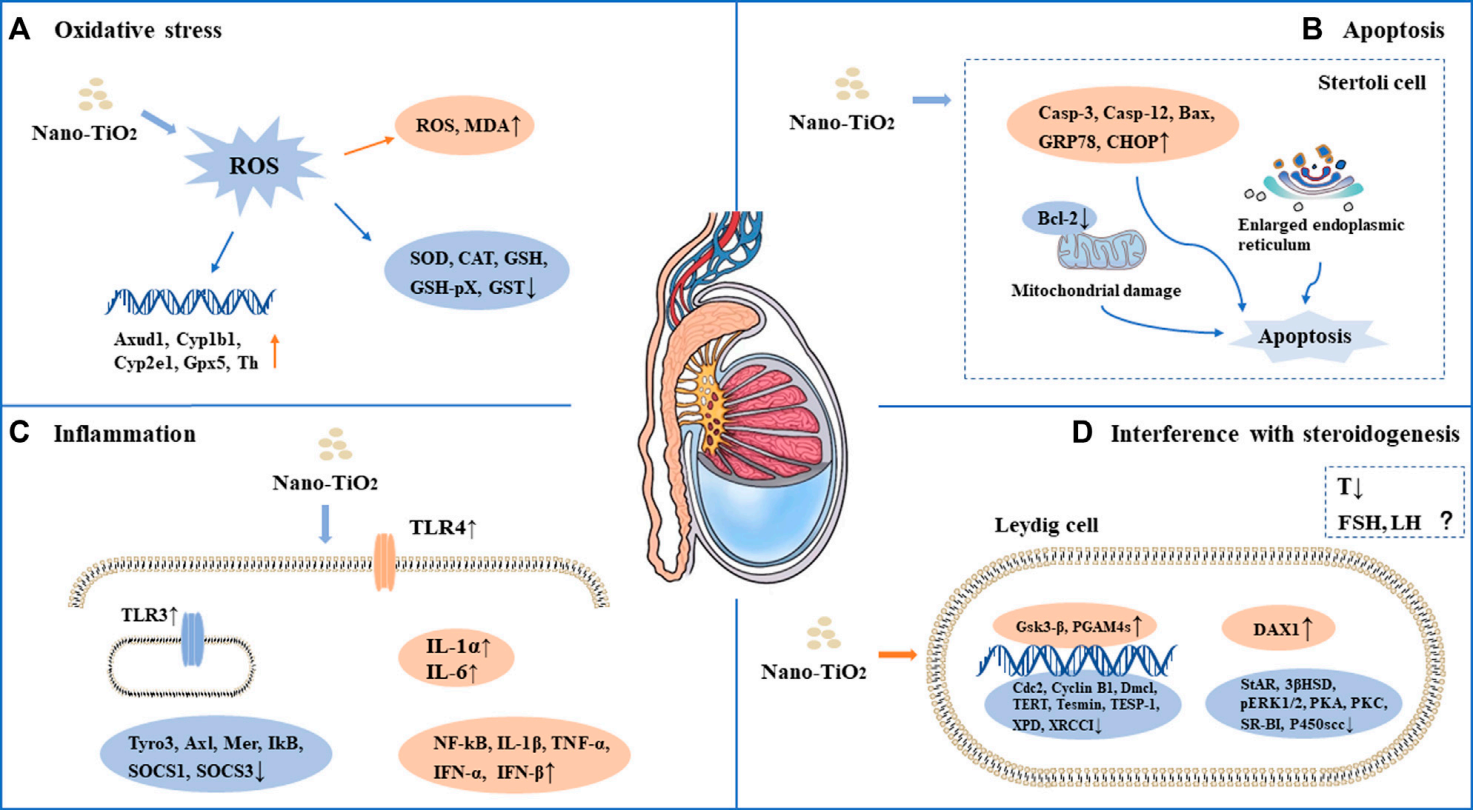
Mechanism of toxicity of nano-TiO_2_ on reproduction in male mammals. **(A)** Oxidative stress. **(B)** Apoptosis. **(C)** Inflammation. **(D)** Interference with steroidogenesis.

As a photocatalytic material, nano-TiO_2_ induces reproductive toxicity directly related to intracellular oxidative stress induced by high-efficiency photocatalysis. Previous studies have suggested that sperm DNA damage caused by nano-TiO_2_ may be related to the direct effect of ROS on genetic material through oxidative stress reactions in cells (Gao et al., 2012; Hong et al., 2015a; Meena et al., 2015; Asare et al., 2016; Hong et al., 2016b; Khorsandi et al., 2017; Morgan et al., 2017; Karimipour et al., 2018; Hussein et al., 2019; Santonastaso et al., 2019; Hong and Zhou, 2020; Ogunsuyi et al., 2020; Danafar et al., 2021; Liu et al., 2021; Mancuso et al., 2021; Halawa et al., 2022; Meng et al., 2022). It has been shown that nano-TiO_2_ can increase the synthesis of 8-oxo-2′-deoxyguanosine, which is a component of DNA, thus causing genetic damage. Gao et al. found mitochondrial swelling, irregularity of the nuclear membrane, nuclear chromatin condensation and margination after exposure to nano-TiO_2_. ROS production increased significantly in the ovary. The results suggest that ovarian apoptosis after nano-TiO_2_ exposure, and ROS accumulation led to apoptosis and DNA peroxidation in the ovary under nano-TiO_2_-induced toxicity(Gao et al., 2012). Karimipour et al. found that the serum malonaldehyde (MDA) level increased significantly in female mice after exposure to nano-TiO_2_(Lauvas et al., 2019). Santonastaso et al. exposed human sperm to nano-TiO_2_ cultured *in vitro* and found that ROS levels were significantly increased in sperm cells (Santonastaso et al., 2019). It was demonstrated that the expression of Axud1, Cyp1b1, Cyp2e1, Gpx5 and Th related to oxidative stress and apoptosis was upregulated after nano-TiO_2_ exposure in mouse testicular tissue (Gao et al., 2013). Recent studies found that ROS, SOD, CAT, GSH, GSH-Px and GST levels were decreased after exposure to nano-TiO_2_ in testicular cells, while MDA levels were increased (Gao et al., 2013; Hong et al., 2015a; Meena et al., 2015; Hong et al., 2016b; Khorsandi et al., 2017; Morgan et al., 2017; Shahin and Mohamed, 2017; Hussein et al., 2019; Hong and Zhou, 2020; Ogunsuyi et al., 2020; Danafar et al., 2021; Mancuso et al., 2021; Halawa et al., 2022; Meng et al., 2022; Li et al., 2023). Oral antioxidants (such as quercetin and chitosan) can reverse the damage of nano-TiO_2_, suggesting that oxidative stress may play an important role in the damage caused by nano-TiO_2_ in the male reproductive system (Khorsandi et al., 2017; Halawa et al., 2022). See in Figure 1A and Figure 2A.

### 4.2 Apoptosis

Apoptosis plays an important role in male spermatogenesis by helping maintain the proper ratio of germ cells to surrounding supporting cells. Studies have shown that nano-TiO_2_ may interrupt the apoptosis process of germ cells. Tassnari et al. found the incidence of apoptosis increased significantly in granulosa cells in the ovary with an increase in the exposure dose of nano-TiO_2_(Tassinari et al., 2014). However, the mechanism was not clarified. Sirotkin et al. demonstrated the inhibitory action of nano-TiO_2_ on markers of mitochondrial/cytoplasmic apoptosis, caspase 3 and bax. This observation suggested that nano-TiO_2_ can directly inhibit ovarian granulosa cell cytoplasmic apoptosis (Sirotkin et al., 2021b). Moreover, suppression of both proliferation and apoptosis indicated that nano-TiO_2_ can suppress ovarian cell turnover directly. Guo et al. showed that intraperitoneal injection of 500 mg/kg/d BW nano-TiO_2_ in male rats could induce germ cell apoptosis (Guo et al., 2009). Hong et al. exposed primary cultured Sertoli cells of ICR mice to different concentrations of nano-TiO_2_ for 24 h and found that the apoptosis rate and death rate of Sertoli cells increased significantly. Upregulation of caspase-3, cytochrome c, caspase-12, Bax, C/EBP homologous protein, and glucose-regulated protein 78 expression and downregulation of bcl-2 protein expression in primary cultured SCs were induced by nano-TiO_2_ treatment (Hong et al., 2016b). Mancuso et al. exposed porcine prepubertal SCs to 100 μg/mL nano-TiO_2_ and noted that several large vacuoles were present in SCs, probably as a result of increased frequency of lipid droplets and/or enlarged endoplasmic reticulum and/or apoptotic mitochondria (Mancuso et al., 2021). At the same time, the caspase-3 pathway was activated, which cleaved p53 into active fragments of p19 kDa, inducing apoptosis. Several studies have shown that long-term exposure to nano-TiO_2_ at low doses can increase the apoptosis rate of SCs and stromal cells in spermatogenic tubules in male testes (Meena et al., 2015; Hong et al., 2016a; Khorsandi et al., 2017; Mao et al., 2017; Shahin and Mohamed, 2017; Li et al., 2023). In conclusion, the induction of apoptosis is one of the important reasons for the damage to the reproductive system caused by nano-TiO_2_. See in Figure 1B and Figure 2B.

### 4.3 Inflammation

Infection or non-infection and inflammation have harmful effects on reproduction within the male reproductive system, which usually manifest as lowered sperm numbers, reduced androgen production, and temporary loss of fertility. Toll-like receptors (TLRs) are expressed in SCs, which play an important role in the innate responses in the testis. Nano-TiO_2_ can induce the release of inflammatory cytokines through the natural immune response receptor family (TLR), thereby mediating chronic inflammation (Hong et al., 2016a). Therefore, the activation of nano-TiO_2_ and TLR receptors should be given sufficient attention. Zhao et al. found that after exposure to nano-TiO_2_, the expression levels of TNF-α, IL-1β, IL-6, Fas and FasL were significantly increased, while the expression levels of IGF-1, LHR, INH-α and GFF-9 were significantly decreased, resulting in chronic inflammation in the ovary (Zhao et al., 2013). Meena et al. found that the average coefficient of testis decreased and inflammatory cell infiltration occurred in testicular tissue after exposure to nano-TiO_2_ for 30 days (Meena et al., 2015). Hong et al. demonstrated that after long-term exposure to nano-TiO_2_ (9 months), a large number of inflammatory cells infiltrated mouse testicular tissue, and the expression of TLR3 and TLR4 was significantly increased, while the expression of Axl, Tyro3, IkB, Mer, SOCS1 and SOCS3 genes and proteins was significantly decreased (Hong et al., 2016a). Nano-TiO_2_ upregulated the expression of TNF-α, IL-1β, NF-kB, IFN-α and IFN-β and caused inflammation in primary cultured SD rat SCs(Ye et al., 2017). In an *in vitro* study, exposure of porcine prepubertal SCs to nano-TiO_2_ induced upregulated expression of the IL-1α and IL-6 genes and stimulated inflammatory and immunomodulatory responses (Mancuso et al., 2021). See in Figure 1C and Figure 2C.

### 4.4 Interference with steroidogenesis

Nano-TiO_2_ exposure interrupts androgen synthesis in male testis. Androgen is synthesized in LCs in the testis. Li et al. demonstrated that nano-TiO_2_ crosses the membrane into the nucleus or cytoplasm, triggering nuclear condensation and cellular vacuolization. LC viability decreased at the same nano-TiO_2_ concentration in a time-dependent manner, and nano-TiO_2_ treatment decreased mitochondrial membrane potential (MMP), testosterone levels, StAR, 3βHSD, pERK1/2, PKA, PKC, SR-BI, and P450scc and upregulated DAX1 in primary cultured rat LCs(Li et al., 2018). Furthermore, Hong et al. found that individual expression of the mRNAs and proteins of testis-specific genes, including TESP-1, Cyclin B1,Cdc2, TERT, Dmcl, Tesmin, XRCCI and XPD, was significantly decreased, whereas PGAM4 and Gsk3-β expression was greatly elevated in testis, which can reduce spermatogenesis in the altered testis-specific gene expression in nano-TiO_2_ exposed male mice (Hong et al., 2015b). See in Figure 1D and Figure 2D.

## 5 Conclusion and perspective

The existing and still growing evidence demonstrates the potential toxic effects of nano-TiO_2_ particles in humans through different exposure ways, including ingestion, injection and inhalation. Human exposure to nano-TiO_2_ relates to, environmental pollution, occupational settings, or certain consumer goods. It may lead to the aggravation of several chronic diseases, such as the neurodegenerative disease Alzheimer’s disease and glomerulonephritis; hence, nano-TiO_2_ may increase the risk of developing tumours or the progression of pre-existing processes of cancer. We can list the recent Commission Regulation (EU) 2022/63 (Official Journal of the European Union, L11/1, 18 January 2022), which has withdrawn TiO_2_ (E 171) as a food additive due to safety concerns to support this. Human exposure to nano-TiO_2_, whether associated with occupational conditions, environmental pollution, or certain consumer products, may affect reproductive function. Studies have shown that nano-TiO_2_ can accumulate in the reproductive organs or tissues through different pathways, affect the development of ovum and sperm and transmit to the next-generation through biological barriers such as the blood-testosterone barrier and the placental barrier (Kyjovska et al., 2013; Hong et al., 2017; Guillard et al., 2020). However, the transport mechanism by which nano-TiO_2_ penetrates biological barriers remains poorly understood. Studies have proven that TLR receptors are expressed in tissues of the human reproductive system, such as the ovary and testis (Hong et al., 2016a). Therefore, studies on the activation of nano-TiO_2_ and TLR receptors should be considered. The accumulation and toxicity of nano-TiO_2_ in germ cells and tissues may be related to particle size, surface coating, exposure concentration and exposure time. Only one study has explored the toxicity of different morphologies of nano-TiO_2_ on the male reproductive system (Liu et al., 2021). This result demonstrated that anatase is more toxic than rutile. Further studies are needed to explore the effects of different morphologies and particle sizes of nano-TiO_2_ on toxicity in the reproductive system to find a method to decrease the toxicity of nano-TiO_2_. In conclusion, the main causes of nano-TiO_2_ toxicity in the reproductive system include oxidative stress, apoptosis, inflammation and interference with steroidogenesis. Further strategies to minimize the environmental and health impacts of nano-TiO_2_ should include the development of environmentally friendly alternatives to nano-TiO_2_ and its efficient recycling. Due to the potential toxicity of nano-TiO_2_, it is necessary to systematically evaluate the toxicity of nano-TiO_2_ in the human reproductive system through large-scale epidemiological studies to further understand its distribution and accumulation in the reproductive system and transport mechanism through biological barriers. Further studies are needed to explore the mechanisms of nano-TiO_2_ deeply, to develop strategies to alleviate cellular damage and to provide theoretical guidance and a basis for safety evaluation and development of nano-TiO_2_ products.
